# Acrodystrophic axonal polyneuropathy with celiac disease: a case report

**DOI:** 10.1186/s13256-021-03171-z

**Published:** 2021-12-18

**Authors:** S. N. Bardakov, Minh Duc Tran, S. V. Lapin, A. N. Moshnikova, E. U. Kalinina, E. G. Bogdanova, A. V. Bolekhan, B. L. Gavriluk

**Affiliations:** 1 S.M. Kirov Military Medical Academy, Akademika Lebedeva Street, 6., Saint Petersburg, 194044 Russia; 2 Peoples’ Friendship University of Russia, Miklukho-Maklaya Street, 6, Moscow, 117198 Russia; 3 Pavlov First Saint Petersburg State Medical University, L’va Tolstogo Street, 6-8, Saint Petersburg, 197022 Russia; 4 Saint-Petersburg State Pediatric Medical University, Litovskaya Street, 2, Saint Petersburg, 194100 Russia

**Keywords:** Trophic disorders, Axonal polyneuropathy, Celiac disease, Membrane plasma exchange, Transglutaminase

## Abstract

**Background:**

Patients with celiac disease present with not only gastrointestinal symptoms but also extraintestinal manifestations such as anemia, osteopathy, dermatitis herpetiformis, and celiac neuropathy. Despite a fairly wide range of celiac neuropathies, we report a case of the acrodystrophic variant of celiac polyneuropathy, which has not been previously described.

**Case presentation:**

A 41-year-old Ukrainian male suffered from symmetric, sensorimotor axonal polyneuropathy and encephalopathy associated with celiac disease, which is characterized by severe trophic disorders in the lower extremities (trophic ulcers, hyperkeratosis, and anhidrosis). Acrodystrophic changes in the lower extremities were due to both neurogenic and direct immunoinflammatory damaging effects. Clinical–electrophysiological dissociation was also noted, which was represented by a gross axonal lesion with the preservation of muscle strength. The absence of enteropathic manifestations was accompanied by the pronounced histological changes in the duodenal mucosa by IIIb stage of Marsh. A gluten-free diet in combination with membrane plasma exchange and intravenous pulse methylprednisolone was prescribed to reduce the severity of sensory disorders and regression of encephalopathy within 7 months.

**Conclusion:**

Celiac disease may be a potential cause of neuropathy and encephalopathy in adult patients. Further immunosuppressive treatment protocols for both intestinal and extraintestinal manifestations of celiac disease are required.

## Background

Celiac disease is a chronic, heterogeneous, autoimmune disorder that occurs in children and adults who have a genetic predisposition to the development of antibody-mediated damage to small intestinal mucosa (and other organs) resulting in gluten sensitivity [1, 2]. The disease is based on the synthesis of immunoglobulin (Ig)A- and IgG-class antigliadin antibodies (AGAs) and deamidated gliadin peptides (anti-DGPs), anti-endomysial (EMAs), and anti-transglutaminase type 2 antibodies [3–5]. Genetic predispositions related to the presence of the HLA-DQ2 and HLA-DQ8 alleles characterize 95% of patients with celiac disease [2]. Based on serological studies, the prevalence of celiac disease is approximately 1.4%. However, according to morphological studies of bowel biopsy samples, the prevalence is 0.7% [6].

The clinical presentation of celiac disease includes not only gastrointestinal symptoms (diarrhea, abdominal pain, and weight loss), but also extraintestinal manifestations including disorders of: the central and peripheral nervous systems; the cardiovascular, endocrine, genitourinary, and musculoskeletal systems; and the skin (herpetiform dermatitis or Dühring’s disease) [1, 7, 8]. These symptoms suggest a downward trend in the occurrence of the classic enteral form and the predominance of atypical and asymptomatic forms [9].

One of the most common extraintestinal manifestations of celiac disease and hypersensitivity is neuropathy [3], which is typical among males approximately 55 years of age [10–12]. In the USA and Europe, the prevalence of neuropathy among patients with celiac disease varies from 4% to 23% [4, 13] in adults and from 0% to 7% in children [14–19]. When compared with the general population, patients with celiac disease have a 2.5-times greater risk of developing the condition [20]. For clinicians, difficulties occur in cases of celiac neuropathy in the absence of intestinal manifestations, which is characteristic in one-third of adult patients [21], and leads to an average of a 9-year delay in diagnosis [3, 22].

The spectrum of gluten neuropathy variants is highly diverse and includes: sensory and sensorimotor symmetric axonal polyneuropathies [23]; polyneuropathy of thin fibers [10]; multifocal acquired motor neuropathy [24, 25]; sensory ganglionopathy (sensory neuronopathy) [26]; autonomic neuropathy (dysautonomia) manifested by postural nausea, syncope, tachycardia, and dizziness [27]; carpal tunnel syndrome [28]; and asymmetric sensory polyneuropathy [10]. Cases of acute axonal demyelinating forms in childhood have also been described [29]. Rarer variants include neuromyotonia, the inclusion of body myositis, and a combination of polyneuropathy and polymyositis [10].

Despite a fairly wide range of celiac neuropathies, the acrodystrophic variant of polyneuropathy has not been previously described.

Therefore, the purpose of this case study is to present the clinical, immunological, and electrophysiological manifestations of axonal acrodystrophic polyneuropathy associated with celiac disease.

## Case presentation

A 41-year-old Ukrainian male [body mass index (BMI) 37.4] presented with complaints of numbness and cramping in the lower extremities, periodic numbness of fingers I–III of both hands, headache, and general weakness that gradually increased over 8 years. Over the past 2 years, the patient noted complete hair loss in the legs, thinning and increased vulnerability of the skin of the lower limbs, and the appearance of limited areas of severe hyperkeratosis on the feet. A callosity on the first toe of the left foot had led to the formation of a long-term, non-healing infected wound that was complicated by gangrene of the terminal phalanx and had led to its amputation.

**Fig. 1:**
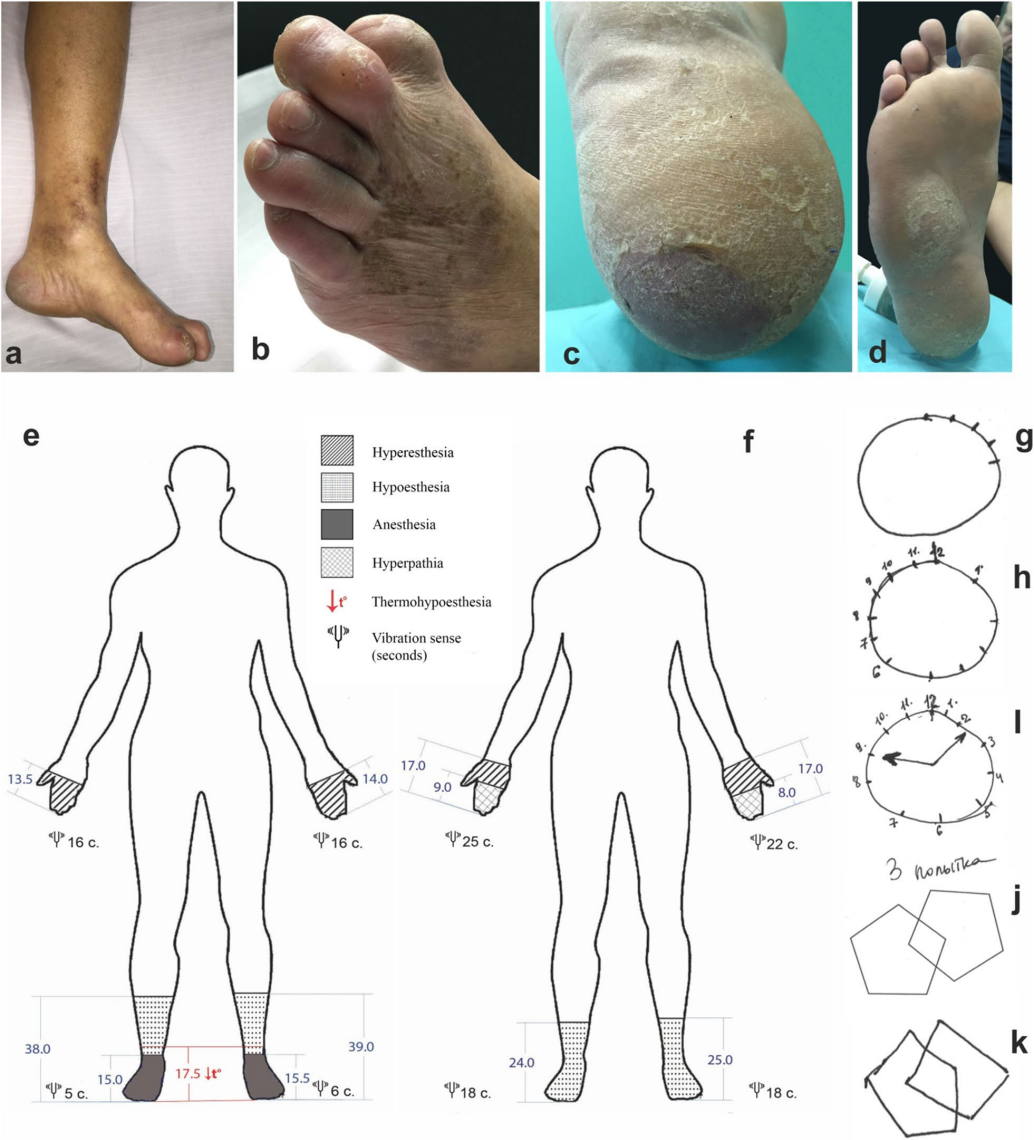
Clinical manifestations of acrodystrophic axonal polyneuropathy of 41-year-old patient

During examination, the skin of both feet was observed to be thinning with pigmentation, lamellar desquamation, and hyperkeratosis on the plantar surfaces (Fig. 1a–d). There were multiple epithelized and unhealed infected wounds on the feet that had developed as a result of microtrauma. The patient also suffered from class II alimentary–constitutional obesity [30, 31].

A neurological examination revealed impaired exteroception with symmetrical hyperesthesia of the metacarpophalangeal joints, hypoesthesia, anesthesia, and thermal hypesthesia to the level of the middle one-third of the legs by polyneuritic type (Fig. 1e). Vibrational sensitivity was reduced to 5 seconds by polyneuritic type. Positional sense was reduced in the distal joints. Tendon reflexes of the lower extremities were weakened. Using the Medical Research Council (MRC) scale, muscle strength in the flexors of the lower legs was reduced by 4 grades, by 5 grades in the extensors, and 5 grades in the distal sections. Before the start of treatment in September 2017, hand dynamometry measured 38 kg and 36 kg on the left and right sides, respectively, which increased to 46 kg and 45 kg following treatment in February 2018. Moderate atrophy and muscle pain in the lower legs and short foot muscles were also found. Sensitive ataxia was noted as well. Autonomic trophic disturbance of the lower extremities was characterized by hyperkeratosis, anhidrosis, and livedo reticularis (Fig. 1a).

Despite the absence of complaints from the patient and his relatives, a decrease in cognition was uncovered using the Montreal Cognitive Assessment (MoCa) scale with a score of 18 out of 30, the Mini-Mental State Exam (MMSE) with a score of 23 out of 30, and the Frontal Assessment Battery (FAB) with a score of 13 out of 18. In addition, tests evaluating spatial orientation (the clock drawing task and drawing complex or three-dimensional figures), attention, delayed recall, and phonetic speech activity were found to cause the most difficulties (Fig. 1g–k).

### Laboratory and instrumental tests

To identify the causes of polyneuritis syndrome, we excluded endocrine diseases [normal indicators of insulin, proinsulin, glycated HbA1c, adrenocorticotropic hormone, thyroid-stimulating hormone (TSH), triiodothyronine (T3), and thyroxine (T4)], infections (cytomegalovirus, Epstein–Barr virus, herpes, and borreliosis), and dysmetabolic origins (vitamin levels for B1, B6, B12, and homocysteine were normal; and amyloid deposition in the subcutaneous fat was absent). Electrophoresis of serum proteins allowed us to exclude the presence of paraprotein with a slight increase in the β1 fraction and hypergammaglobulinemia. Among the possible autoimmune causes, tests for antineutrophil cytoplasmic antibodies (ANCA), antinuclear factor, and extractable nuclear antigen antibodies were negative. An immunoblot of antibodies to gangliosides and onconeural antigens was negative as well. However, recombinant tissue transglutaminase 2 (TG2) IgA antibodies were found to be five times the normal level (90.3 IU/ml, which is normally less than 20 IU/ml). Anti-DGP IgG antibody values were normal (22.8 IU/ml).

Among all the classes of antibodies, only the IgE level was increased at 122 IU/ml.

As an additional source of confirmation for celiac disease, the HLA-DQ2 and HLA-DQ8 alleles were found to be present.

No pathologies or abnormalities were detected following an electrocardiogram, echocardiography, thyroid, abdominal and pelvis ultrasounds, head magnetic resonance imaging (MRI), and chest computed tomography (CT). However, during a nerve conduction study (NCS), signs of gross axonal damage to the motor and sensory fibers of the lower extremities was uncovered, with a complete block at the distal stimulation points of the left tibial and peroneal nerves and signs of secondary demyelination. In the upper limbs, there were signs of moderate axonopathy of the ulnar nerve (Table 1).

**Table 1:** Results of NCS of a 41-year-old male before and following multidisciplinary therapy

Date	July 2017			February 2018		
Parameters	Amplitude, мB	Velocity, m/second	Residual latency, m/second	Amplitude, мB	Velocity, m/second	Residual latency, m/second
Motor NCSs						
Ulnar nerve	4.7/7.5	N	1.6/2.1	5.8/5.4	N	1.7/–
Median nerve	11.8/10.2	N	1.3/1.2	13.0/11.0	N	–/–
Tibial nerve	0.2/0.0	22.3/31.5	1.5/5.7	0.4/0.0	20.5/27.2	4.1/4.9
Peroneal nerve (extensor digitorum brevis muscle)	0.2/0.0	36.2/33.0	5.9/6.2	0.2/0.0	48.1/49.2	6.5/5.8
Peroneal nerve (tibialis anterior muscle)	6.2/4.9	48.9/47.5	–	5.4/4.4	50/60	–
Peroneal nerve (peroneus longus muscle)	7.0/8.1	47.8/47.1	–	7.4/7.3	54/45.7	–
Sensory NCSs						
Ulnar nerve	12.1/15.2	55/44	–	17.6/2.7	58.0/53.7	–
Median nerve	1.4/1.2	59.1/48.8	–	9.5/8.7	56.1/52.1	–
Sural nerve	1.3/1.1	33.7/35.5	–	0.7/0.8	41/40	–
Superficial peroneal nerve	0/0	0/0	–	–	–	–

F waves	Minimum latency	Minimum latency
Ulnar nerve	24.1	25.0
Median nerve	27.1	25.2

**Fig. 2:**
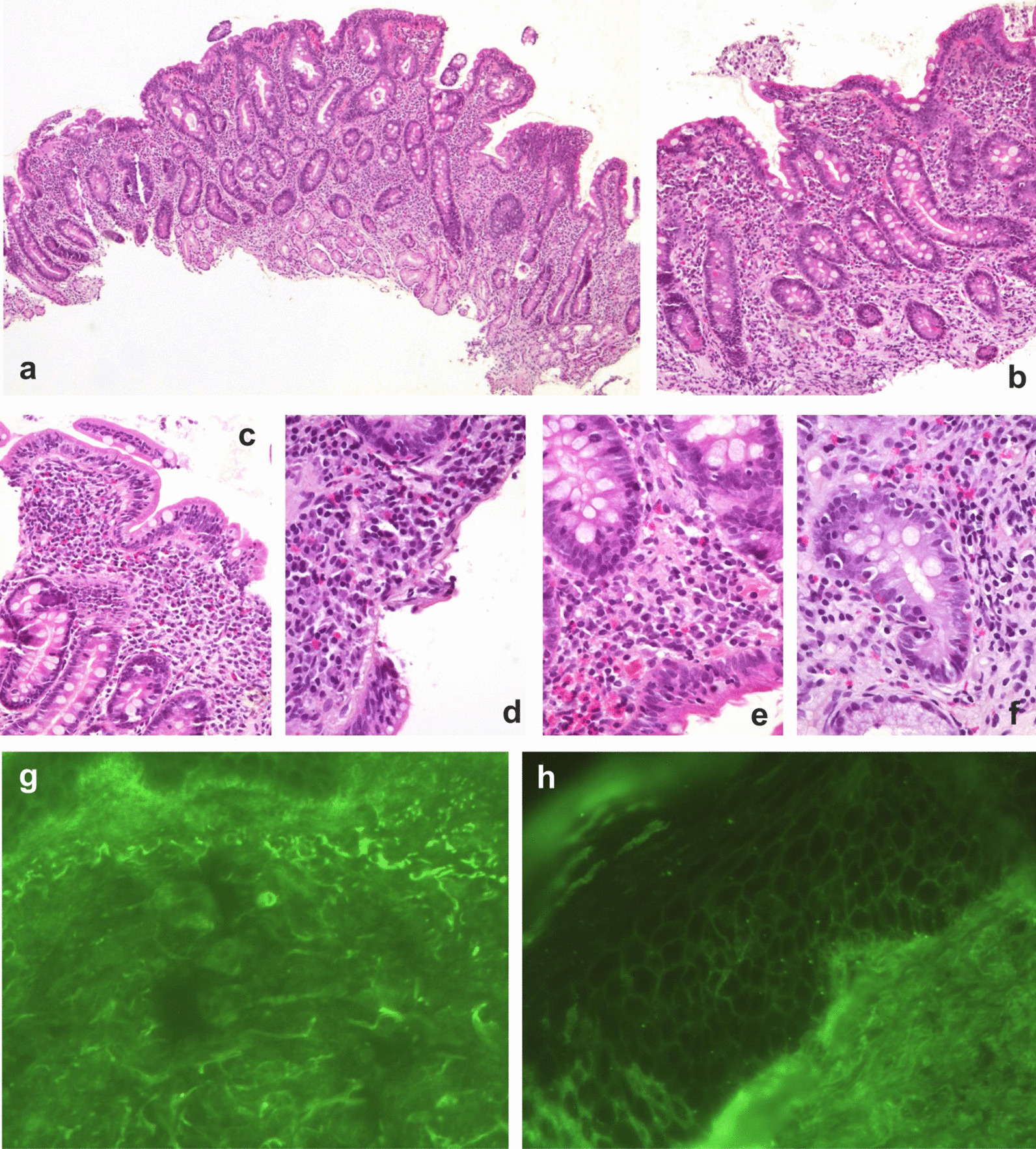
Pathohistomorphological manifestations the duodenum and the lower one-third of the shins of 41-year-old patient

To assess the involvement of the gastrointestinal pathological process, a fibroesophagogastroscopy was performed, during which an erythematous gastroduodenopathy was revealed. Biopsies of the gastric and duodenal mucosa were sent for morphological study (Fig. 2).

Morphological analysis of the duodenal mucosa biopsy (Table 2, Fig. 2) identified changes (subatrophy of villi in combination with crypt hyperplasia) that correspond to celiac disease according to the Marsh IIIB classification.

**Table 2:** Morphometric study of a duodenal biopsy

Parameter	Size *M ± SD*
Total thickness of the mucous membrane, microns	391.2* ± *17.2
Height of the villi (VH), microns	213.1* ± *12.4
Width of the villi, microns	128.1* ± *10.1
Crypt depth (CD), microns	321.7* ± *3.1
Crypt width, microns	54.3* ± *0.3
VH/CD ratio	0.7* ± *0.1
Height of the villus enterocytes (HVE), microns	34.1* ± *0.1
Crypt enterocytes (HCE), microns	8.2* ± *0.1
HVE/HCE coefficient	4.1* ± *0.01
Number of intraepithelial lymphocytes per 100 enterocytes	43* ± *5
Degree of fibrosis (+/±/−)	+
Lymphoplasmacytic infiltration	+
Edema	+

*VH* Height of the villi,* CD* Crypt Depth,* HVE* Height of the villus enterocytes,* HCE* Height of crypt enterocytes,* M±SD* Mean ± standard deviation

Figure 2 shows duodenal mucosa relief, atrophy of the villi, and deepening of the crypts. In Fig. 2a, inflammatory infiltration, reactive changes in epithelial cells are presented. In Fig. 2b and c, pronounced diffuse lymphoplasmacytic infiltration, an increase of intraepithelial lymphocytes, and a decrease of goblet cells in the surface sections can be seen. Figure 2d and e shows pronounced diffuse lymphoplasmacytic infiltration, with a sharp increase in intraepithelial lymphocytes. In Fig. 2f, there are reactive changes in epithelial cells with nucleo- and nucleolomegaly, perinuclear vacuolization, nuclear hyperchromia, and increased mitotic activity. From Fig. 2a to f, the plates were stained with hematoxylin and eosin. Examination using direct immunofluorescence (DIF) reaction using antisera (Fig. 2g) uncovered C1q deposition (2+) in the papillary dermis, and linear IgA deposition (1+) along the basement membrane of the epidermis (Fig. 2h).

Examination of the skin biopsy by DIF using antisera uncovered C1q deposits in the papillary layer of the dermis in the lower one-third of the shin, minor IgA deposits at the intercellular contacts of the epidermis, and linear deposits along the basement membrane of the epidermis. The pattern was similar to linear IgA dermatosis.

### Results of treatment

Multidisciplinary therapy was initiated that included two cycles of five operations of medium-volume membrane plasma exchange, with an exfusion volume of 25–30% of the circulating plasma volume in combination with 1 g of intravenous pulse methylprednisolone. Metabolic therapy included the administration of α-lipoic acid (1200 mg/day); vitamins B6 (50 mg/day), B12 (1000 mg/day), and E (400 units/day); ipidacrine (60 mg/day); sulodexide (500 IU/day); and l-carnitine (3000 mg/day), as well as the initiation of gluten-free diet (GFD).

Eight months following the start of treatment, regression of the clinical symptoms of axonal polyneuropathy and cognitive deficiency was observed (Fig. 1f). During the next neurological examination, polyneuritic hypoesthesia was observed at the level of the lower third of the legs with full normalization of thermoception and vibrational sensation. In the upper limbs, hyperesthesia (to the level of the wrist joints) in the distal phalanges occurred, and signs of hyperpathy appeared. The trophic status of the cutaneous lower extremities returned to normal, and previously nonhealing wounds epithelized. Cognitive function was normalized (MMSE score of 28; FAB score of 18; and a MoCA score of 28). The level of recombinant TG2 IgA antibodies decreased to 13.0 IU/ml.

## Discussion and conclusions

The clinical symptoms of symmetric axonal sensorimotor polyneuropathy were an extraintestinal manifestation of celiac disease, as was the encephalopathy detected during subsequent examinations. The uncommon features that differentiated this case included pronounced trophic disorders (nonhealing trophic ulcers, hyperkeratosis, and anhidrosis), and the absence of clinical manifestations of enteropathy in the presence of gross morphological changes in the duodenal mucosa. Clinical–electroneuromyographic dissociation was also noted, which was represented by a significant decrease in the amplitudes of the M waves of the lower-extremity nerves with complete muscle-strength conservation in the distal regions. Together, these features caused a diagnostic delay of 8 years.

Gluten neuropathy is one of the most common extraintestinal manifestations of celiac disease and gluten hypersensitivity and usually occurs in males in their early fifties. In this case, however, the manifestation occurred much earlier in a 31-year-old [3, 10, 11]. Neuropathy was the only manifestation of celiac disease, which is in line with described cases of peripheral nerve damage that occurs before and after the development of intestinal manifestations, as well as in isolation [10, 21].

The gradual development of symmetric hypoesthesia in the lower extremities corresponded to the most common chronic course of gluten neuropathies [3, 10, 23], which is in contrast to the more rare, acute forms described in children [19, 29, 32]. The ascending character of hypoesthesia, followed by impaired vibration sensitivity, made it possible to classify this variant as symmetric, sensorimotor polyneuropathy involving length-dependent neuropathy, which is the second most common after polyneuropathy of thin fibers [10, 14, 15, 21]. Despite the duration of disease in this case, there was no muscle weakness, which has been reported in 10% of cases of sensorimotor axonal forms [10, 11, 21]. In the presence of NCS signs of axonopathy of sensory fibers, a pronounced dissociation was observed between the absence of muscle weakness and the presence of gross axonal damage to the motor fibers, with a complete block of the peroneal and tibial nerves in the distal regions.

Possible causes of polyneuropathy based on the idea of malabsorption [10] due to celiac disease were excluded by assessing the levels of vitamins B1, B6, and B12 and folic acid. In addition, antibodies to gangliosides (which are observed in 65% of cases [21] due to the presence of molecular mimicry mechanisms between gliadin epitopes and nerve antigens) were not detected [10, 33].

Gluten neuropathies can also be associated with ataxia, dysarthria, myoclonus, extrapyramidal, cognitive, and autonomic disorders [10, 12]. In our case, sensitive ataxia was observed due to impaired vibration sensitivity, which occurs in 26% of cases of celiac polyneuropathy [28].

An examination of the patient at 38 years of age revealed signs of slowly progressing encephalopathy, which was primarily represented by mild cognitive impairment and personality changes not previously observed by the patient or his relatives. The combined development of polyneuropathy and cognitive impairment (chronic and rapidly progressive) has been previously described in patients at an average age of 46.9 years [3, 28, 34], although there have been reports of dementia-like syndromes, characterized by significant variability during childhood and adolescence [35–37]. It should be noted that encephalopathy, which occurs in 21% of celiac disease cases, is more often associated with enteropathy, in contrast to neuropathy and cerebellar ataxia [22].

In our case, encephalopathy presented with mild frontal dysfunction (a FAB score 13 out of 18 points), mild cognitive impairment (including decreased attention and memory for separated events, and visual-spatial orientation disorder), which is consistent with reports from the literature [36, 37]. These changes correspond to the front-subcortical type [34], with no headache, acalculia, and diffuse or focal changes in the brain according to MRI results [3, 34].

During the DIF reaction of the patient's serum with cryosections of the cerebellum and cerebral cortex of rats, no pathological types of luminescence were detected, indicating the presence of autoreactive antibodies. Damage to the nervous system in celiac disease is due to the presence of TG2 in the smooth muscle layer of cerebral vessels, as well as the presence of antibodies to type 6 tissue transglutaminase (TG6) in the cerebellum [22, 38, 39].

In this case, there was five times the normal level in the level of recombinant TG2 IgA antibodies, which was likely due to their decrease when the diet therapy for alimentary obesity had begun, as antibodies may be present for 6–12 months after the start of diet therapy [3]. Anti-DGP (IgG and IgA) antibodies were within normal limits. EMAs were not available for investigation; therefore, a biopsy of the distal duodenum was performed to verify the diagnosis [3, 40].

The development of neurological manifestations in the absence of clinical signs of enteropathy was observed in our 41-year-old patient, and is also observed in one-third of adult celiac disease and gluten hypersensitivity cases [3]. Moreover, our morphological study revealed signs of chronic atrophic duodenitis with grade III crypt hyperplasia and erosion, which corresponds to stage IIIb of the Marsh histological classification of Marsh–Oberhuber [7]. Similar dissociation has also been observed in 94.2% of asymptomatic children with high titers of anti-TG (IgA) antibodies in the II–III stages of Marsh [40]. Therefore, the diagnosis of latent (silent) celiac disease was confirmed based on a combination of a positive test for anti-TG-IgA, morphological changes in the duodenum that corresponded to the Marsh II–III classification stages, and the detection of HLA-DQ2 and HLA-DQ8 antigens [3, 6, 40].

One distinct feature of this case was the pronounced trophic changes in the skin, which caused the formation of long-healing ulcers following minor trauma. This feature was considered to be a manifestation of the insufficiency of neurogenic trophic influences. However, DIF performed in a skin biopsy revealed an autoimmune factor. Bullous dermatitis was absent in this patient, but in the skin biopsy specimen a primary deposition of C1q complement was observed in the papillary layer of the dermis (2+) where TSH type 3 was located (a high-affinity antibody that determines the development of herpetiform dermatitis or Dühring’s disease). However, IgA deposits were minimal at the border of the papillary dermis along the basement membrane and in the intercellular contacts of the epidermis, which corresponded to the localization of TSH type 2 [41]. Typical herpetiform Dühring dermatitis is characterized by itchy papules and the absence of obvious intestinal symptoms [42] and is caused mainly by the presence of antibodies to TSH type 3. These determine the presence of granular IgA deposits in the area of the basement membrane between the epidermis and dermis with complement C3 deposition during DIF [43]. Changes in the skin had a linear form of IgA deposition, but, unlike linear bullous dermatosis, there were no characteristic vesicular, erythematous papules or itchy skin [8, 41, 44]. Some authors consider this type of dermatosis to be associated with celiac disease [45]. Due to the complement with mediated lesions of the dermis, prolonged nonhealing wounds are probable, and not only due to neurogenic trophic disorders.

Seven months after the start of a strict GFD, and with the addition of short-term immunomodulation (two cycles of membrane plasma exchange and intravenous pulse methylprednisolone), a decrease in the severity of sensitivity disorders, normalization of the trophic status of the skin, and complete regression of encephalopathy were observed. Concurrently, a sevenfold decrease in the level of antibodies to TSH type 2 was noted, which is in line with reports concerning the efficacy of diet therapy [2, 16]. The effectiveness of a strict GFD does not depend on the presence or absence of enteropathy [22]. Moreover, the reported effectiveness of a therapeutic apheresis is variable. In particular, the positive effect of the treatment for neuromyelitis optica and herpetiform dermatitis had been shown [46]. In contrast, the lack of an effect of therapeutic apheresis has been observed in a patient with cognitive impairment and celiac disease [34], as well as in three cases of multifocal neuropathy [21]. There are studies showing unchanged levels of antibodies to TSH type 6 and gliadin during a GFD [3, 21, 39]. However, the positive effects of intravenous Ig [24], as well as the presence of incomplete regression of the clinical manifestations of neuropathy following implementation of the diet [3], substantiate the use of membrane plasma exchange to accelerate and increase the effectiveness of therapy.

In conclusion, sensorimotor axonal polyneuropathy with severe trophic disorders may be one of the variants of celiac neuropathy and is due to neurogenic autoimmune factors. Celiac disease is a heterogeneous, autoimmune disease and may be a potential cause of neuropathy and encephalopathy in adult patients. With cases of incomplete regression or refractoriness during a gluten-free diet in patients, further immunosuppressive treatment protocols for both intestinal and extraintestinal manifestations of celiac disease are required.

## Data Availability

Not applicable.

## Abbreviations

AGA: Anti-gliadin antibody
ANCA: Antineutrophil cytoplasmic antibody
BMI: Body mass index
CD: Crypt depth
CT: Computed tomography
DGP: Deamidated gliadin peptide
DIF: Direct immunofluorescence
EMA: Endomysial antibody
FAB: Frontal Assessment Battery
GFD: Gluten-free diet
HCE: Height crypt enterocytes
HLA: Human leukocyte antigen
HVE: Height of the villus enterocytes
MMSE: Mini-Mental State Exam
MoCa: Montreal Cognitive Assessment
MRC: Medical Research Council
MRI: Magnetic resonance imaging
NCS: Nerve conduction study
T3: Triiodothyronine
T4: Thyroxine
TG: Transglutaminase
TSH: Thyroid-stimulating hormone
VH: Height of the villi
